# ID 78 - Custo-Efetividade das Terapias Direcionadas ao Eixo do Receptor Androgênico (ARATs) de Segunda Geração no Câncer de Próstata Resistente à Castração (CPRC)

**DOI:** 10.5327/2237-9622.2025.v34s1.78

**Published:** 2025-11-25

**Authors:** Maiara Silva Araujo, Layssa Andrade Oliveira, Ludmila Peres Gargano, Haliton Alves de Oliveira, Rosa Camila Lucchetta

**Keywords:** análise custo-benefício, avaliação da tecnologia biomédica, análise de sobrevida, neoplasias da próstata, técnicas de apoio para a decisão

## Abstract

**Introdução:**

O câncer de próstata é a segunda neoplasia mais comum entre homens globalmente. O tratamento é baseado no estadiamento, histopatologia, prognóstico e características do paciente. A quimioterapia (QT) é indicada para pacientes com progressão da doença apesar do uso de terapia de privação androgênica (TPA) e a abiraterona para pacientes com uso prévio de quimioterapia. Nos últimos anos, outros antiandrogênios conhecidos como agentes direcionados ao eixo do receptor de andrógeno (ARAT – do inglês, ) de segunda geração - enzalutamida, apalutamida e darolutamida – foram desenvolvidos. Assim, este estudo analisou a custo-utilidade dos ARATs de segunda geração em câncer de próstata resistente em três populações clínicas não metastático (CPRCnm), metastático (CPRCm) em pacientes virgens de QT, e CPRCm com uso prévio de QT, comparados às opções recomendadas no Sistema Único de Saúde (SUS).

**Método:**

Foram conduzidas análises de custo-utilidade, utilizando modelos de sobrevida particionada. As estimativas de sobrevida global e sobrevida livre de progressão ou sobrevida livre de metástase foram extraídas dos estudos primários e extrapoladas para um horizonte de dez (CPRCm em pacientes virgens de QT e com uso prévio de QT) ou 20 (CPRCnm) anos utilizando diferentes distribuições paramétricas. As curvas foram selecionadas com base em critérios de informação (AIC/BIC) e inspeção visual. Para CPRCm, as curvas foram ajustadas com os HR da comparação indireta entre abiraterona e enzalutamida. Valores de utilidade foram obtidos da literatura internacional para obtenção de resultados em anos de vida ajustados pela qualidade (AVAQ). Para análise da incerteza, análises de sensibilidade determinísticas e probabilísticas foram realizadas.

**Resultados:**

Para CPRCnm, a darolutamida teve a menor razão de custo-efetividade incremental (RCEI), com R$204.350/AVAQ ganho, seguida da enzalutamida (R$362.995/AVAQ ganho) e apalutamida (R$598.780/AVAQ ganho), não sendo custo-efetivas no limiar de R$120.000 por AVAQ ganho. Para CPRCm em pacientes virgens de QT, tanto abiraterona quanto enzalutamida mostraram maiores benefício clínico e custo total em relação à TPA isolada. No entanto, a abiraterona foi considerada custo-efetiva (RCEI de R$36.759/AVAQ ganho), enquanto a enzalutamida não (RCEI de R$702.849/AVAQ ganho). Para CPRCm em pacientes com uso prévio de QT, a enzalutamida apresentou maior benefício clínico, porém a um custo total superior, comparada tanto à TPA isolada quanto à abiraterona. A enzalutamida não foi custo-efetiva (R$1.052.095/AVAQ ganho comparada à abiraterona; R$394.563/AVAQ ganho comparada à TPA). Nas análises de sensibilidade, essas terapias apresentaram benefício clínico com custo incremental, exceto para CPRCm em pacientes com uso prévio de QT, onde a enzalutamida comparada à abiraterona apresentou possibilidade de menor efetividade e maior custo.

**Conclusão:**

As avaliações econômicas indicaram que a incorporação de apalutamida, darolutamida e enzalutamida no SUS não seriam custo-efetivas para CPRC. Apenas a abiraterona foi considerada custo-efetiva para pacientes com CPRCm virgens de QT.

